# Prioritization of HCV treatment in the direct-acting antiviral era: An economic evaluation

**DOI:** 10.1016/j.jhep.2016.02.007

**Published:** 2016-07

**Authors:** Natasha K. Martin, Peter Vickerman, Gregory J. Dore, Jason Grebely, Alec Miners, John Cairns, Graham R. Foster, Sharon J. Hutchinson, David J. Goldberg, Thomas C.S. Martin, Mary Ramsay, STOP-HCV Consortium, Matthew Hickman

**Affiliations:** 1 Division of Global Public Health, University of California San Diego, San Diego, USA; 2 School of Social and Community Medicine, University of Bristol, UK; 3 Kirby Institute, UNSW Australia, Sydney, Australia; 4 Faculty of Public Health and Policy, London School of Hygiene and Tropical Medicine, UK; 5 Queen Mary’s University of London, UK; 6 Glasgow Caledonian University, UK; 7 Health Protection Scotland, UK; 8 Guy’s and St Thomas’s NHS Foundation Trust, London, UK; 9 Public Health England, UK

**Keywords:** HCV, hepatitis C virus, PWID, people who inject drugs, PegIFN, pegylated interferon, RBV, ribavirin, IFN-free, interferon-free, DAA, direct-acting antiviral, HCC, hepatocellular carcinoma, QALY, quality adjusted life-year, NMB, net monetary benefit, ESLD, end stage liver disease, WTP, willingness to pay, Prevention, People who inject drugs, Hepatitis C, Treatment

## Abstract

**Background & Aims:**

We determined the optimal HCV treatment prioritization strategy for interferon-free (IFN-free) HCV direct-acting antivirals (DAAs) by disease stage and risk status incorporating treatment of people who inject drugs (PWID).

**Methods:**

A dynamic HCV transmission and progression model compared the cost-effectiveness of treating patients early *vs.* delaying until cirrhosis for patients with mild or moderate fibrosis, where PWID chronic HCV prevalence was 20, 40 or 60%. Treatment duration was 12 weeks at £3300/wk, to achieve a 95% sustained viral response and was varied by genotype/stage in alternative scenarios. We estimated long-term health costs (in £UK = €1.3 = $1.5) and outcomes as quality adjusted life-years (QALYs) gained using a £20,000 willingness to pay per QALY threshold. We ranked strategies with net monetary benefit (NMB); negative NMB implies delay treatment.

**Results:**

The most cost-effective group to treat were PWID with moderate fibrosis (mean NMB per early treatment £60,640/£23,968 at 20/40% chronic prevalence, respectively), followed by PWID with mild fibrosis (NMB £59,258 and £19,421, respectively) then ex-PWID/non-PWID with moderate fibrosis (NMB £9,404). Treatment of ex-PWID/non-PWID with mild fibrosis could be delayed (NMB -£3,650). In populations with 60% chronic HCV among PWID it was only cost-effective to prioritize DAAs to ex-PWID/non-PWID with moderate fibrosis. For every one PWID in the 20% chronic HCV setting, 2 new HCV infections were averted. One extra HCV-related death was averted per 13 people with moderate disease treated. Rankings were unchanged with reduced drug costs or varied sustained virological response/duration by genotype/fibrosis stage.

**Conclusions:**

Treating PWID with moderate or mild HCV with IFN-free DAAs is cost-effective compared to delay until cirrhosis, except when chronic HCV prevalence and reinfection risk is very high.

## Graphical abstract

**Figure:**
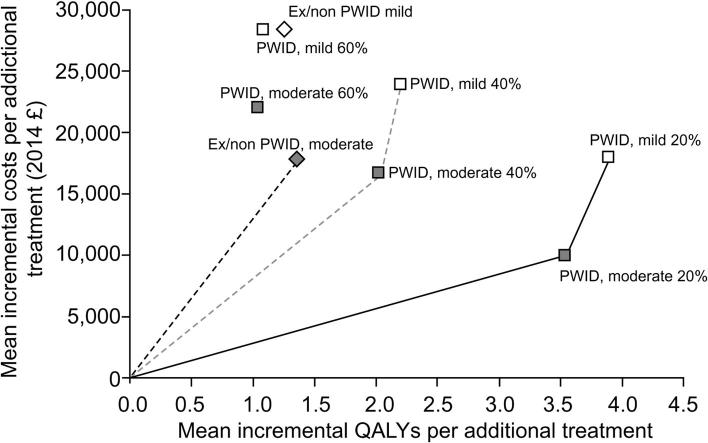

## Introduction

Chronic infection with hepatitis C virus (HCV) is a leading cause of morbidity and mortality across the world. Globally, an estimated 80–150 million people are chronically infected with HCV, which if left untreated can lead to cirrhosis, liver cancer, and death [1], [2]. In high-income countries, people who inject drugs (PWID) are the main risk group for HCV transmission, contributing to >90% of new infections in settings such as the UK [3].

Effective antiviral treatments for HCV can result in a sustained virological response (SVR, equating to a cure) in the large majority of people [4]. HCV antiviral treatment could also be a key component in preventing HCV transmission through the reduction of chronic HCV prevalence among PWID [5], [6], [7]. Previous research has indicated that treating PWID with interferon (IFN)-containing therapy (i.e., pegylated interferon (PegIFN) and ribavirin (RBV)) is likely to be more cost-effective than treating non- or former-PWID with no ongoing risk behavior due to the substantial potential prevention benefit [8]. Current HCV treatment rates in many countries, however, are insufficient to reduce either the rise in end stage liver disease (ESLD) [9], [10] or HCV transmission [11].

The HCV antiviral treatment landscape is rapidly changing. SVR rates with new IFN-free direct-acting antivirals (DAAs) are higher than for PegIFN + RBV: at >90% for all genotypes compared to <50% for genotype 1 and up to 80% for genotype 3 [4]. Crucially, IFN-free DAA treatment has improved SVR in people with genotype 1 cirrhosis from ∼30% to >80% [12], [13]. IFN-free DAAs are highly tolerable, oral-only, shorter duration (12–24 weeks) and will likely involve once daily regimens. These new therapies are associated, however, with considerable treatment costs (e.g., $60,000–80,000 per 12 week course).

Although some new DAA agents have been deemed cost-effective in the UK [14] and are reimbursable in the US and Australia, there is heated debate as to how best to prioritize patients for treatment [15], [16]. International guidelines in 2014 suggest prioritizing IFN-free DAA therapy for patients with advanced liver disease [17], [18]. A recent analysis from the United States demonstrated that IFN-free DAA treatment among people with mild stages of fibrosis (F0 or F1) is not cost-effective compared to delaying treatment until more moderate stages of fibrosis (F2) [19]. However, these recommendations are based on expected individual patient-level benefit in reduced progression to ESLD, and neglect potential prevention benefits to the population due to the impact on HCV transmission [16].

We therefore use a dynamic model of HCV transmission among PWID, combined with data on disease progression, and treatment to determine the more cost-effective strategy for prioritizing HCV antiviral treatment after diagnosis.

## Methods

### Mathematical model

An existing dynamic cost-effectiveness model of HCV transmission, disease progression, and treatment was adapted [8] (Supplementary Fig. 1). The model incorporated HCV transmission among PWID, such that HCV infection and reinfection was related to the background prevalence of chronic infection among PWID, which could change over time. The model included the risk of reinfection after treatment for PWID, and also the population benefits of reducing onward transmission. We used the model to examine three chronic HCV prevalence settings among PWID (20%, 40% and 60%) at baseline. This corresponded to baseline incidences of infection/reinfection among PWID of 4% (2.5–97.5% Confidence Interval (95% CI) 3–5%), 9% (95% CI 7–13%), and 21% (95% CI 15–30%), in the 20%, 40%, and 60% chronic prevalence scenarios, respectively. The model was open, with PWIDs entering the population on initiation of injecting and were tracked after permanent cessation of injecting when they were assumed to be no longer at risk of reinfection or transmission.

The model was a deterministic, compartmental model which was stratified by risk status (PWID, former-PWID), HCV genotype (genotype 1 and 4, genotype 2, and genotype 3) and infection status and disease stage (never infected or infected and spontaneously cleared, mild HCV, moderate HCV, compensated cirrhosis [CC], decompensated cirrhosis [DC], hepatocellular carcinoma [HCC], liver transplant, post-transplant). For simplicity, we assumed an individual had one dominant genotype strain which affected treatment SVR, and that an individual’s risk of acquiring a specific genotype was related to the circulating prevalence of each genotype. Additionally, for those stages eligible for antiviral treatment (mild HCV, moderate HCV, and compensated cirrhosis), the model was further stratified by treatment status (never treated, on treatment, SVR, non-SVR). Those who achieved SVR were at risk of reinfection; we assumed no change in risk behavior after treatment, so each individual’s risk of reinfection was equal to that of primary infection. We assumed that those with mild or moderate fibrosis who achieved SVR were at no risk of further liver disease progression unless they were reinfected. Based on clinical evidence, we assumed that those with compensated cirrhosis who achieved SVR remained at elevated risk of disease progression due to existing liver damage [20], [21]. Individuals who did not attain SVR proceeded through the natural history of liver disease progression and were assumed to be ineligible for retreatment, as no drugs are currently licensed for retreatment of IFN-free DAA failures. The base-case assumed the risk of transmission or acquisition of HCV was independent of disease stage or duration of injecting, as evidence is unclear whether, apart from the first year, injecting risk increases or decreases over the course of an injecting career.

### Antiviral treatment scenarios

We explored three antiviral treatment scenarios to assess whether differences in the characteristics of the treatment course affected the prioritization strategy.

1. Future IFN-free DAA scenario: IFN-free DAAs for 12 weeks with 95% SVR for all disease stages and genotypes [4], [22], [23], [24]. We used this scenario as the base-case for most of our analyses.
2. ‘Current’ DAA scenario: IFN-free DAAs with 90–95% SVR for mild/moderate HCV, and 70–90% SVR for compensated cirrhosis depending on genotype. Treatment durations are 8–12 weeks (genotypes 1 and 2) and 24 weeks (genotype 3).
3. ‘Current’ DAA scenario except PegIFN/RBV for mild G3: As in scenario (2) but with PegIFN/RBV for mild genotype 3.

Assumptions regarding SVR and treatment durations, and costs for different HCV antiviral treatment regimes can be found in Table 1. As future costs of many IFN-free regimens are not yet determined, we assumed a weekly drug cost of £3300 per week (cost of sofosbuvir + ledipasvir [25]). Treatment delivery costs assumed are £90 per week [26] for ex/non-PWID, PWID delivery is 120% of non/ex-PWID cost [8]. Treatment delivery costs included the costs of staff time and tests/investigations; we assumed higher treatment delivery costs for PWID due to additional staff time and psychiatric assessments as in previous economic evaluations [8], [27], [28]. In the sensitivity analysis, we varied the SVR by disease stage and genotype, cost, and treatment duration.

### Prioritization analysis using cost-effectiveness methods

For each level of chronic HCV prevalence in PWID (20%, 40% and 60%), we compared the following treatment options to assess the most cost-effective prioritization strategy:

#### Baseline

Treat everyone with compensated cirrhosis (mainly ex-PWID with no ongoing risk so have very little ‘treatment as prevention’ benefit) every year. We chose this baseline to represent current guidance and the real-world prioritization of treating individuals with advanced disease (CC) first. We did not treat individuals with DC, HCC, or post-transplant, as treatment for these groups is recommended on a case-by-case basis and disease progression outcomes are still uncertain; individuals who are not in these stages at baseline were all treated at the CC stage upon progression.

#### Intervention

In addition to baseline treatment of all those with CC, we modeled treating, in addition, each year for the next 10 years:

• 1 PWID (in our population of 1000 PWID) at the mild stage
• 1 PWID (in our population of 1000 PWID) at the moderate stage
• 1 non or ex injector at the mild stage
• 1 non or ex injector at the moderate stage

As shown in previous work including population-level treatment as prevention benefits [8], [27], the cost-effectiveness of treatment was strongly dependent on the treatment rate. The higher the treatment rate for PWID, the greater the prevention benefits, and therefore the greater the cost-effectiveness of treatment for PWID. Therefore, we conservatively examined a very low treatment rate among PWID because HCV treatment rates among PWID are extremely low in the UK and most other global settings (<1% PWID per year) and so it does not overly bias towards treatment of PWID.

We calculated the costs and quality adjusted life-years (QALYs) for a further 40 years, giving a total time horizon of 50 years.

The cost-effectiveness analysis used a UK health care provider perspective. Costs were valued in 2014 UK pounds (£1 = €1.3 = $1.50 USD) and health outcomes were expressed in QALYs. Both costs and health utilities were discounted at 3.5% per annum in the base-case according to UK National Institute for Health and Care Excellence (NICE) guidelines [29].

Uncertainty in the underlying parameters was accounted for, such that epidemiological parameters, disease transition probabilities, costs, and health benefits were analysed using multivariate random sampling from appropriate distributions. For each of the 1,000 sampled parameter sets, we simulated three chronic HCV baseline prevalence scenarios among PWID at equilibrium, which represented the range of prevalence observed across most sites in Europe and other developed countries, (20%, 40%, and 60%), obtaining matched simulations for each prevalence setting and treatment scenario. These simulations therefore provided endemic infection population numbers in each disease category (PWID and ex/non-PWID) given a total population of 1,000 PWID.

Using the sampled 1,000 simulation sets, we calculated the mean incremental costs and mean incremental QALYs gained from an intervention treatment strategy compared to delayed treatment until compensated cirrhosis. All those who were untreated received best supportive care with its associated costs. We then ranked strategies through calculating the net monetary benefit (NMB) through the equation NMB = ((mean incremental QALYs × willingness to pay threshold) – mean incremental costs), using £20,000 and £30,000 willingness to pay per additional QALY (WTP) thresholds for the UK, with the highest rank having the highest NMB. If strategies were similar in NMB, we assessed the probability that one is more cost-effective than another using the runs from the probabilistic sensitivity analysis. Patient groups with a negative NBM (<£0) were not prioritized. A negative NMB implied that treatment should be delayed in the patient group until a later disease stage – and that treatment at that stage was not cost-effective at a specific WTP. We also plotted cost-effectiveness efficiency frontiers on the cost-effectiveness plane; interventions which lie off the frontier were dominated (more expensive and/or gaining fewer QALYs). Additionally, we presented the impact on HCV-related mortality, cases of end stage liver disease (decompensated cirrhosis and HCC), and on new HCV infections. Using the full set of 1,000 probabilistic runs, we utilized one-way sensitivity analyses to test how changes in the model structure or individual parameter assumptions impact the order in which the DAAs should be prioritized.

### Transition probabilities

HCV natural history disease stage probabilities are shown in Table 2. Based on HCV progression values, the model predicted 12% [95% CI 7–20%] cirrhosis by 20 years. New PWID entered the model at 20 years old and had an elevated risk of mortality due to drug-related death during injecting (1% per year [30]), as compared to ex/non-PWID (average lifespan 76 years [31]). PWID permanently ceased injecting after an average of 11 years (sampled from 6 to 16 years due to substantial uncertainty in this estimate [32], [33]).

### Utilities

Health utilities were attached to each disease stage and obtained from previous UK economic evaluations and the UK mild HCV trial (Table 2). We assumed that uninfected PWID experience a lower quality of life compared to an uninfected ex/non-PWID. For the base-case, we further assumed that HCV-infected health utilities are comparable between PWID and ex/non-PWID, due to an absence of evidence indicating differences between these populations. This resulted in a smaller quality of life loss upon HCV infection for PWID, therefore the individual-level benefit of treating a PWID was less than for a non-injector.

### Costs

Health care provider costs for untreated HCV disease stages were taken from previous economic evaluations and the UK mild HCV trial, and inflated to 2014 GBP using the Hospital and Community Health Services Pay and Prices Index (Table 2).

### Sensitivity analysis

We performed a multivariate probabilistic uncertainty analysis for the base-case treatment scenario (‘Future IFN-free DAAs’, 95% SVR) to assess the sensitivity of the NMB to uncertainty in the underlying parameters. We also performed a number of one-way sensitivity analyses on the following parameters: time horizon (20 or 100 years, compared to 50 in base-case), discount rate (0% and 6% discounting, compared to 3.5% in base-case), IFN-free DAA drug costs (at one-quarter (£825), one-half (£1650), or double (£6600) per week compared to base-case), PWID SVR rate (reduced by a relative 10% compared to non/ex-IDU SVR, equal in base-case), SVR with cirrhosis (reduced to 85% from 95% in base-case), behavior after treatment (50% reduction in risk after treatment compared to no reduction), average injecting duration (5 or 20 years, compared to a mean of 11 years in base-case). Additionally, because future costs are uncertain and likely to reduce over time, we simulated an additional scenario where DAA costs reduced by 75% in 10 years (from £3300 to £825/week (approximately $1200 USD/week), comparable to the price of sofosbuvir/ledipasvir available in some low/middle income countries.

## Results

### Prioritizing IFN-free DAAs for all genotypes with 95% SVR

**Fig. 1:**
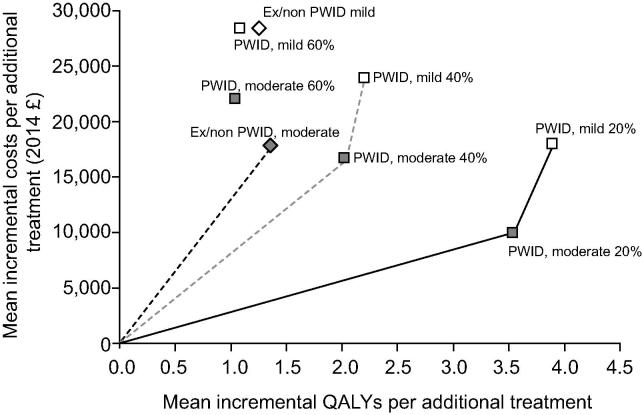
**Results on the incremental cost-effectiveness plane showing the efficient frontiers for 20% (solid line), 40% (dashed grey line), and 60% (dashed black line) chronic prevalence scenarios for the ‘Future IFN-free DAA’ treatment scenario.** Results shown for treatment of PWID populations (squares) and ex-non-injectors (diamonds) with moderate disease (grey squares) and mild disease (white squares) compared to delayed treatment at the compensated cirrhosis stage. Treatment scenarios which do not fall on the frontier are dominated (more expensive with fewer benefits). Costs/QALYs are incremental to treatment of all those with compensated cirrhosis and best supportive care for all others.

For a 20% and 40% baseline chronic prevalence among PWID, the most cost-effective prioritization strategy was to target PWID before ex/non-PWID due to the substantial prevention benefits of treatment (Table 3, Fig. 1) and early treatment of PWID was cost-effective under a £20,000 WTP (Table 4, Supplementary Table 1). Using a £20,000 WTP and in the scenario with 20% chronic prevalence among PWID, the most cost-effective group to treat were moderate PWID (mean NMB per additional early treatment £60,640) in 54% of the simulations and mild PWID in 46% of simulations (mean NMB £59,258). Treatment of mild or moderate ex/non-PWID dominated (i.e., was more expensive and results in fewer QALY gains). However, if all PWID were treated, the next best target group would be ex- or non-injectors with moderate disease (mean NMB £9,404). Compared to delaying treatment, treatment of mild ex/non-PWID is not cost-effective under this WTP (mean NMB -£3,650). Similarly, at 40% chronic prevalence (Table 3, Fig. 1), targeting PWID was the most cost-effective strategy, with moderate and mild PWID yielding similar benefit to each other (mean NMB £23,868 and £19,421 for the moderate and mild PWID, respectively) but less benefit was achieved overall than in the 20% prevalence scenario where greater prevention benefits were accrued (HCV infections averted).

By contrast, at 60% chronic prevalence (Table 3, Fig. 1, Supplementary Table 1), targeting moderate ex/non-PWID was the only cost-effective option compared to delaying treatment. In this setting, due to the very high risk of reinfection, treatment of PWID was not cost-effective compared to delay.

### Impact on new HCV infections averted

Treatment of PWID averted the most new infections in the lower prevalence scenarios; for example at 20% chronic prevalence, 2.3 [95% CI 2–2.6] new HCV infections were averted per early treatment of mild PWID. In the higher prevalence scenarios, fewer infections were averted due to higher reinfection rates (treatment of mild PWID averts 0.78 [95% CI 0.75–0.81] and 0.23 [95% CI 0.21–0.24] new HCV infections per early treatment in the 40% and 60% prevalence scenarios respectively). Treatment of moderate PWID averted slightly fewer infections due to the elevated risk of mortality from disease progression.

### Impact on HCV mortality and end stage liver disease burden per treatment

The strategy that had the greatest impact on HCV mortality and burden of end stage liver disease was treatment of people with moderate disease (Table 3). Compared to delayed treatment at cirrhosis, each early treatment of a moderate ex-non-PWID in the low HCV prevalence setting averted 0.08 HCV deaths (95% CI 0.03–0.17), 0.06 (95% CI 0.03–0.11) cases of decompensated cirrhosis, and 0.04 (95% CI 0.01–0.13) cases of HCC over 50 years, i.e., for every 13 people treated with moderate disease, one HCV-related death was prevented. ESLD outcomes were similar for treatment of moderate PWID in prevalence settings below 60% (Table 3). Early treatment at the mild stage had less impact on immediate disease outcomes; treating a mild ex-non-PWID averted 0.03 (95% CI 0.01–0.05) HCV deaths, 0.02 (95% CI 0.01–0.04) cases of decompensated cirrhosis, and 0.01 (95% CI 0–0.04) cases of HCC per treatment over 50 years. Treatment of PWID with moderate disease averted fewer deaths and cases of ESLD in the 60% prevalence scenarios compared to treatment of ex/non-PWID due to the immediate risk of reinfection (Table 3).

### Alternative DAA treatment scenarios

The prioritization rankings remain unchanged across different treatment availability scenarios, such as using ‘Current’ DAA treatments (with lower SVR for cirrhosis and longer treatment durations for genotype 3), or the ‘Current’ DAA scenario with IFN/RBV for mild genotype 3 as is currently recommended by NICE in the UK (Table 4). Therefore, the prioritization rankings were robust to differences in treatment characteristics across genotypes. Importantly, however, treatment of all groups was cost-effective (positive NMB) for these scenarios. The increase in cost-effectiveness of treatment at the mild stage in these scenarios was driven by the substantial increase in SVR between the mild/moderate and cirrhosis stages.

### Sensitivity analysis on ‘Future IFN-free DAAs’ treatment scenario

Based on NMB with a £20,000 WTP, the prioritization of patients for treatment were sensitive to assumptions in a few analyses (Supplementary Table 2). Variations in cost of DAAs did not change the relative prioritization rankings, but did alter the cost-effectiveness of early treatment. If the cost of DAA treatment was halved or reduced by 75% then treatment of all groups becomes cost-effective compared to delaying treatment (positive NMB), though the rankings remain unchanged. Similarly, if DAA prices reduce by 75% in 10 years, the prioritization rankings and cost-effectiveness (positive or negative NMB) for each group remained unchanged, however early treatment of all groups became slightly less cost-effective compared to delayed treatment at cirrhosis (Supplementary Table 2). This was because our intervention examined the impact of a 10-year early treatment program (with costs/utilities followed for a total of 50 years), so with early treatment, fewer individuals are treated later (after 10 years) when prices drop as they have already been treated earlier with higher priced DAAs. If a shorter (20 year) time horizon is adopted, then treatment should be delayed (negative NMB) for all except moderate PWID as few individual benefits are accrued due to the long timescale of liver disease progression, and if the discount rate is increased to 6% then treatment of ex-non-injectors should be delayed.

Prioritization and ranking were unchanged in relation to different SVRs in PWID (reduced 10% compared to ex-non-PWID) or in people with cirrhosis (reduced to 85% from 95%). Rankings were also insensitive to changes in risk behavior after treatment (e.g., 50% reduction in risk), or varying the average injecting duration (5 years or 20 years), though greater NMB were achieved in settings with shorter injecting durations as more infections were averted despite achieving lower prevalence reductions (results not shown).

## Discussion

Based on NMB alone, which ranked patient groups in terms of cost of treatment and QALYs gained, PWID with chronic HCV in the UK (and other sites with <40% chronic HCV among PWID) would be prioritized ahead of other patients with moderate disease, after treating people with severe disease, due to the additional benefit of averting secondary infections. In contrast, treatment of other patients (non-PWID) with mild disease is unlikely to be cost-effective at current HCV drug prices using a willingness to pay (WTP) threshold of £20,000 per QALY compared to delaying treatment. We estimate that, in sites with a chronic HCV prevalence of 20% among PWID, treating one person with new HCV direct-acting antivirals (DAA) prevents more than two other infections. Treating people with moderate disease earlier (before cirrhosis) also could avert end stage liver disease (ESLD) and HCV-related deaths, at a rate of 0.08 (95% CI 0.03–0.17) HCV deaths per person treated over a fifty year period i.e., for every 13 (6–34) people treated one extra HCV death may be averted. Our prioritization results were robust to variations in SVR/duration by genotype and fibrosis stage. Variations in cost of DAAs did not change the relative prioritization rankings, but did alter the absolute cost-effectiveness of early treatment, such that cheaper DAAs increased the cost-effectiveness of treatment, but a drop in DAA prices in the future made early treatment less cost-effective.

### Limitations

Interpretation of our model projections needs to take into account a number of limitations. First, despite the promise that new HCV treatments will transform the treatment landscape, specific parameters related to IFN-free DAA regimens (costs, utilities, and health outcomes) are uncertain. The model results were robust, however, across the scenarios examined, and, although the decision to delay treatment was preferred (negative NMB) in some scenarios (influenced by drug cost, time horizon, or WTP threshold) the relative rankings remained the same throughout the sensitivity analysis. Therefore, although DAA costs may reduce in the future, and cost-effectiveness will vary, the relative prioritization of a treatment at a given time with a given cost will remain the same regardless of the absolute price.

Second, there are limited data on SVR of IFN-free DAA in different patient groups in real-world settings. Given the uncertainty in SVR rates between earlier disease stages and cirrhosis, we assumed equal SVR for the base-case scenario which would not overly favour early treatment. Earlier research suggests that SVR in PWIDs can be comparable to other patient groups, but these studies are small, focus on the old treatments, and are subject to selection bias [34]. Reducing SVR for PWID in our model from 95% to 85% in people with moderate or mild disease, however, did not alter the ranking of patients for treatment and early treatment remained cost-effective.

Third, the model assumes that all PWID with chronic infection are equally likely to be tested and treated, and we did not model any heterogeneity in transmission risk or access to treatment among PWID. It is plausible that high risk individuals (who are at greater risk of transmitting infection to others) are currently less likely to access HCV treatment than those at lower risk and therefore, that projections of the prevention benefit of treatment may be over-estimated. We think such an event, however, is unlikely as other analyses have shown that heterogeneity in injecting risk and treatment uptake has little effect on the model projections as long as there is minimal movement between populations of injectors at high and low risk [5].

Fourth, our model was parameterized to the UK and may not be generalizable to low or middle income country settings. In other countries, the costs and availability of care for each of the HCV disease stages are likely to vary – for example, in many settings liver transplantation may not be an option. The health utilities may not be applicable to other settings, as different populations may apply different weights to mild/moderate disease *vs.* cirrhosis. Additionally, we did not include HIV-HCV coinfection in the model as coinfection rates are low (<1%) in the UK, but many countries have a high burden of coinfection and this population could have different health utilities and health care usage.

Finally, in our model we assumed no transmission occurs among other (non-PWID) patients and therefore settings where other routes of transmission (such as iatrogenic transmission) are common, also may be able to show greater prevention benefits through early treatment of non-PWID who are at risk of transmission [35].

### Comparison with existing studies

There is an extensive body of literature examining the cost-effectiveness of various HCV treatment regimens for PWID incorporating individual-level benefits only [8], [28], [36], [37], [38], [39], [40], [41], [42], [43]. Where examined, most of these analyses conclude it is more cost-effective to prioritize treatment for those with more advanced liver disease, as those with mild stage disease may not all otherwise progress to more severe disease. A recent analysis found that early DAA treatment at a mild stage (F0 or F1) was not cost-effective at a $50,000 WTP compared to delay until F2, assuming a treatment cost of $100,000 [19]. Our analysis similarly found treatment of mild ex/non-PWID was not cost-effective in contrast to delaying treatment. However, in contrast we have found substantial benefits in most scenarios for treating mild PWID due to accrued prevention benefits (which are not modeled and taken into account by these other studies or recent NICE appraisals of the new DAA treatments [14]). The 2015 European Association of the Study of the Liver (EASL) Clinical Guidelines [44] now recommend that “treatment should be prioritized regardless of the fibrosis stage for individuals at risk of transmitting HCV, including active injection drug users…” but with no supporting evidence for this change in recommendation since 2014 [17]. Our economic models provide an evidence base for the guidance but only for populations with low to moderate chronic HCV prevalence among PWID (below 40%).

### Implications

Clinicians and policy-makers face a dilemma. New DAAs offer highly effective treatments but at a considerable price. In the UK there are over 100,000 people with chronic HCV, and in the USA, up to 3 million. With so many patients, and only 5,000 treatments per year in England, even “cost-effective” treatments will need to be rationed in order to manage the health budget. The first priority will be to manage and treat people with severe disease, which is our baseline strategy. The clinical and policy decision is then who next to offer treatment to. Delaying treatment for people with mild disease is an attractive option – but fails to take account of the prevention benefits of HCV treatment. Clinical decision-making surrounding treatment incorporates numerous factors, including individual, population, ethical and economic considerations. However, we have shown that from an economic standpoint, decisions surrounding clinical guidance should take into account both disease stage and current risk status. HCV treatment as a public health prevention strategy, however, requires treatment scale-up in order to show observable reductions in HCV prevalence and transmission, which is likely to happen only once the costs of DAAs are reduced.

## Financial support

NKM acknowledges research funding from the National Institute for Drug Abuse (R01 DA037773-01A1) and the UCSD Center for AIDS Research, an NIH-funded program (P30 AI036214), which is supported by the following NIH institutes and centers: NIAID, NCI, NIMH, NIDA, NICHD, NHLBI, NIA, NIGMS, and NIDDK. NKM, AM, PV, and GRF are members of the STOP-HCV consortium, which is funded by the Medical Research Council (MR/K01532X/1). NKM, PV, and MH are supported by the NIHR Health Protection Research Unit on Evaluation of Interventions. The work was partly funded by Health Protection Scotland and Common Services Agency Scotland. The Kirby Institute is funded by the Australian Government Department of Health and Ageing. JG is supported by a National Health and Medical Research Council Career Development Fellowship. The views expressed are those of the authors and not necessarily those of the UK NHS, UK NIHR, UK Department of Health, or the Australian Government.

## Conflict of interest

NKM has received research grants from Gilead unrelated to this work and has received honoraria from AbbVie, Gilead, and Jannsen. JG is a consultant/advisor and has received research grants from AbbVie, Bristol-Myers Squibb, Gilead, Janssen, and Merck. GJD is an advisory board member and receives honorarium from Roche, Merck, Janssen, Gilead, Bristol-Myers Squibb, AbbVie, has received research grant funding from Roche, Merck, Janssen, Gilead, Bristol-Myers Squibb, Vertex, Boehringer Ingelheim, AbbVie, and travel sponsorship from Roche, Merck, Janssen, Gilead, and Bristol-Myers Squibb. GRF has received speaker and consultancy fees from AbbVie, Gilead, BMS, Janssen, and MSD. SH has received consultancy fees from AbbVie, Gilead, Janssen, MSD, Roche, and research grants from Janssen.

## Authors’ contributions

NKM, PV, AM, and JC and MH designed the analysis. NKM built the model, performed the simulations, and drafted the manuscript. All authors contributed towards the interpretation of the data and manuscript editing, approved the final version, and agree to be accountable for the work.

## Figures and Tables

**Table 1:** **Model assumptions regarding antiviral treatment sustained viral response (SVR) rates for the different treatment scenarios examined.**

∗As future costs of many IFN-free regimens are not yet determined or known, we assume a weekly cost equal to that of sofosbuvir + ledipasvir (approximately £3300 per week [25]) but vary this (one-quarter, one-half, and double the baseline cost) in the sensitivity analysis. Treatment delivery costs assumed are £90 per week [26] for ex/non-PWID, PWID delivery is 120% of non/ex-PWID cost [8]. Treatment delivery costs include the costs of staff time and tests/investigations; we assume higher treatment delivery costs for PWID due to additional staff time and psychiatric assessments as previous economic evaluations [8], [27], [28].

**Table 2:** **Model parameters for transition rates, health utilities, and disease stage costs.**

^‡^PPI = Hospital and Community Health Services Pay and Prices Index inflation factor from 03/04 for 13/14 (1.29). CC, compensated cirrhosis; HCC, hepatocellular carcinoma; LT, liver transplant; SVR, sustained viral response; PegIFN, pegylated interferon; RBV, ribavirin.

**Table 3:** **Net monetary benefit of prioritizing early IFN-free DAA treatment (95% SVR all genotypes) to different groups compared to delayed treatment until compensated cirrhosis.**

NR, not ranked as net monetary benefit negative; HCC, hepatocellular carcinoma.

**Table 4:** **Net monetary benefit using the ‘Current DAA’ treatment scenario and the ‘Current DAA with IFN/RBV for mild genotype 3’ treatment scenarios with a £20,000 WTP.**

The treatment SVR and duration assumptions for these scenarios can be found in Table 1.
