# Supplementary material for: Prioritization of HCV treatment in the direct-acting antiviral era: An economic evaluation
Source: J Hepatol. 2016 Jul;65(1):17–25. doi: 10.1016/j.jhep.2016.02.007 (PMC4914770; doi:10.1016/j.jhep.2016.02.007)
Supplement: Supplementary Fig. 1 and Tables 1 and 2 [file mmc1.pdf]

Prioritization of HCV treatment in the direct-acting antiviral era: An economic evaluation

Natasha K Martin, Peter Vickerman, Gregory J Dore, Jason Grebely, Alec Miners, John Cairns, Graham R Foster, Sharon J Hutchinson, David J Goldberg, Thomas C.S. Martin, Mary Ramsay, the STOP-HCV Consortium, Matthew Hickman

Table of contents

Supplementary Fig. 1 ……………………..……………………………………..……………2

Supplementary Table. 1………………………………………………………………………..3

Supplementary Table. 2………………………………………………………………………..4

(A)

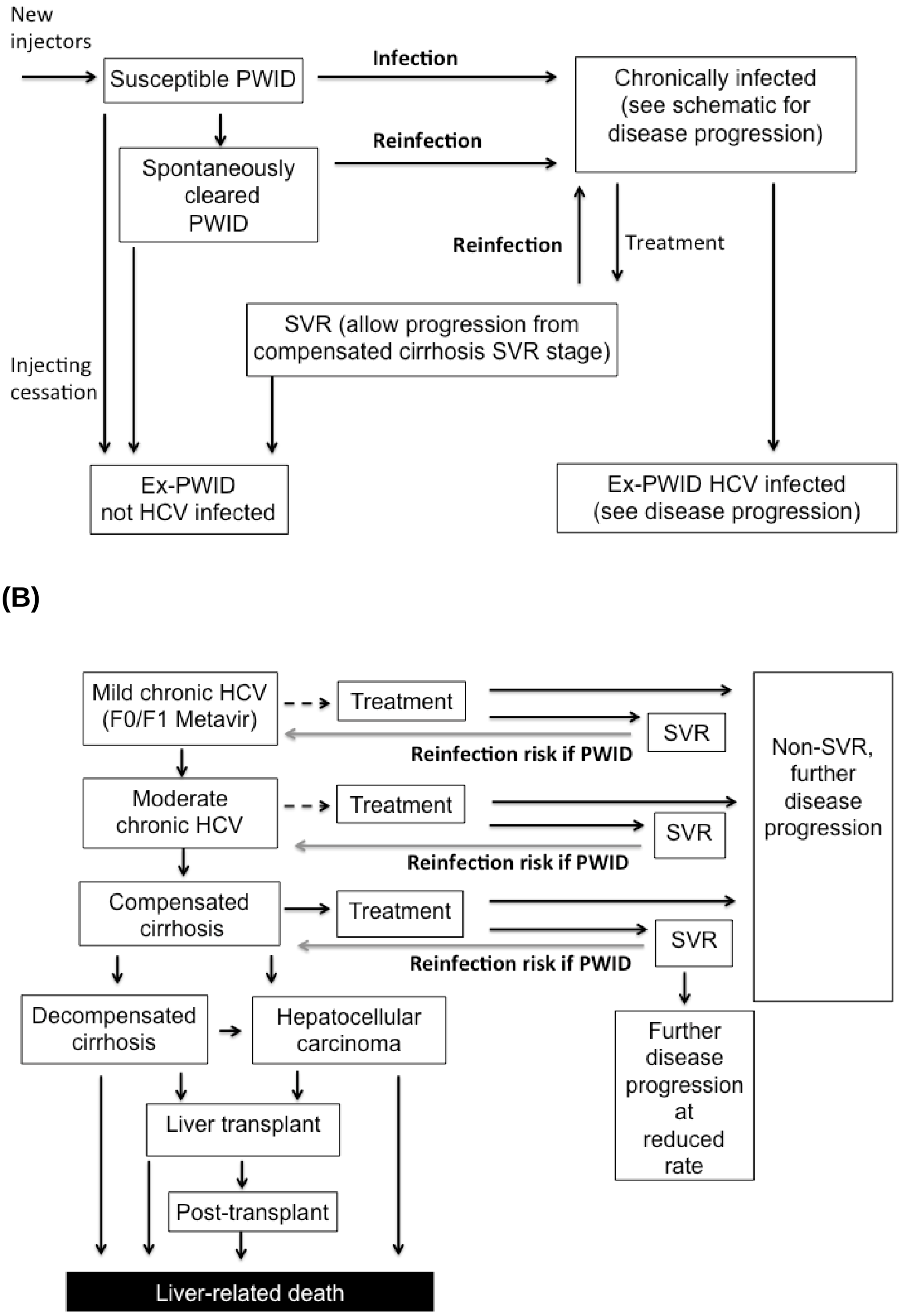

Supplementary Fig 1. (A) Model schematic of PWID and ex-PWID populations and (B) HCV disease progression. The model is also stratified by genotype. Background mortality occurs from all stages and we incorporate elevated drug related mortality among PWID. Gray lines denote transitions for PWID only. Dashed lines denote treatment scenarios compared in the analysis.

Supplementary Table 1. Mean incremental cost-effectiveness ratios (ICERs) for the base-case scenario.

	**Mean ICER***
Ex/non-PWID, mild	£22,932
Ex/non PWID, moderate	£13,081
PWID, mild 20% chronic prevalence	£4,656
PWID, moderate 20% chronic prevalence	£2,855
PWID, mild 40% chronic prevalence	£11,080
PWID, moderate 40% chronic prevalence	£8,242
PWID, mild 60% chronic prevalence	£26,382
PWID, moderate 60% chronic prevalence	£21,284

*ICERs are calculated by dividing the mean incremental costs by the mean incremental QALYs for each scenario compared to a baseline of treatment at the compensated cirrhosis stage.

Supplementary Table 2. Sensitivity analysis of net monetary benefit per early treatment with a £20,000 WTP.

	**Base-case scenario NMB (Rank)**	**50% reduction in risk after treatment**	**PWID SVR reduced by 10% compared to ex/non-PWID**	**SVR reduced to 85% for cirrhosis**	**Average injecting duration 5 years**	**Average injecting duration 20 years**	**20 year time horizon**	**100 year time horizon**	**0% discounting costs/health benefits**	**6% discounting costs/health benefits**	**Quarter DAA**	**Halved DAA costs**	**Double DAA costs**	**DAA costs reduce by 75% in 10 years**
**Ex/non-PWID, mild**	-3,650	-3,650	-3,650	-3,248	-3,650	-3,650	-21,211	3,540	36,400	-12,549	18,421	11,064	-18,364	-5,983
**Ex/non PWID, moderate**	9,404	9,404	9,404	11,127	9,404	9,404	-10,470	16,193	57,950	-2,953	25,032	19,823	-1,015	3,303
**PWID, mild 20% prev**	59,258	58,753	49,198	59,041	78,777	27,470	-5,640	94,558	223,078	19,830	78,480	72,072	46,443	54,269
**PWID, moderate 20% prev**	60,640	60,809	50,817	62,186	81,691	30,003	2,138	90,363	210,216	23,390	74,636	69,971	51,309	52,939
**PWID,**	19,421	20,939	13,711	19,337	22,378	6,371	-12,941	31,890	97,556	322	40,315	33,351	5,492	16,157

**mild 40% prev**														
**PWID, moderate 40% prev**	23,868	26,449	18,023	24,942	27,955	10,821	-5,662	34,468	97,326	4,973	39,984	34,612	13,123	18,216
**PWID, mild 60% prev**	-6,879	-3,329	-9,732	-6,930	-7,264	-11,497	-20,768	-2,102	23,979	-13,766	15,355	7,944	-21,701	-8,780
**PWID, moderate 60% prev**	-1,326	3,530	-4,440	-770	-1,583	-6,012	-14,293	2,791	29,452	-8,928	16,757	10,730	-13,381	-5,187
